# Multifunctional Metal–Organic Frameworks for Enhancing Food Safety and Quality: A Comprehensive Review

**DOI:** 10.3390/foods14234111

**Published:** 2025-11-30

**Authors:** Weina Jiang, Xue Zhou, Xuezhi Yuan, Liang Zhang, Xue Xiao, Jiangyu Zhu, Weiwei Cheng

**Affiliations:** 1 School of Chemistry and Bioengineering, Nanjing Normal University Taizhou College, Taizhou 225300, China; 20181012@nnutc.edu.cn (W.J.); 20081087@nnutc.edu.cn (X.Z.); 20051037@nnutc.edu.cn (X.Y.); 20131005@nnutc.edu.cn (L.Z.); 2 School of Food Science and Engineering, Yangzhou University, Yangzhou 225127, China; mz120232138@stu.yzu.edu.cn (X.X.); 008051@yzu.edu.cn (J.Z.)

**Keywords:** metal-organic frameworks, contaminant detection, sensors, food packaging, decontamination

## Abstract

Food safety and quality are paramount global concerns, with the complexities of the modern supply chain demanding advanced technologies for monitoring, preservation, and decontamination. Conventional methods often fall short due to limitations in speed, sensitivity, cost, and functionality. Metal–organic frameworks (MOFs), a class of crystalline porous materials, have emerged as a highly universal platform to address these challenges, owing to their unprecedented structural tunability, ultrahigh surface areas, and tailorable chemical functionalities. This comprehensive review details the state-of-the-art applications of multifunctional MOFs across the entire spectrum of food safety and quality enhancement. First, the review details the application of MOFs in advanced food analysis, covering their transformative roles as sorbents in sample preparation (e.g., solid-phase extraction and microextraction), as novel stationary phases in chromatography, and as the core components of highly sensitive sensing platforms, including luminescent, colorimetric, electrochemical, and SERS-based sensors for contaminant detection. Subsequently, the role of MOFs in food preservation and packaging is explored, highlighting their use in active packaging systems for ethylene scavenging and controlled antimicrobial release, in intelligent packaging for visual spoilage indication, and as functional fillers for enhancing the barrier properties of packaging materials. Furthermore, the review examines the direct application of MOFs in food processing for the selective adsorptive removal of contaminants from complex food matrices (such as oils and beverages) and as robust, recyclable heterogeneous catalysts. Finally, a critical discussion is presented on the significant challenges that impede widespread adoption. These include concerns regarding biocompatibility and toxicology, issues of long-term stability in complex food matrices, and the hurdles of achieving cost-effective, scalable synthesis. This review not only summarizes recent progress but also provides a forward-looking perspective on the interdisciplinary efforts required to translate these promising nanomaterials from laboratory research into practical, real-world solutions for a safer and higher-quality global food supply.

## 1. Introduction

### 1.1. The Global Challenge of Food Safety and Quality

Ensuring the safety and quality of the global food supply is one of the most significant challenges of the 21st century. The modern food supply chain, a complex network spanning from farm to table, is susceptible to contamination at numerous stages, including production, processing, packaging, and transportation [1]. Food safety has become a paramount global public concern, with recurrent outbreaks of foodborne illnesses posing severe risks to human health and inflicting substantial economic losses worldwide [2]. These risks stem from a wide array of contaminants, including pesticide residues from agricultural practices, veterinary drugs used in animal husbandry, mycotoxins produced by fungal growth, heavy metals from environmental pollution, pathogenic microorganisms, and illegal food additives [3,4].

With increasing globalization, food products are distributed across international borders, amplifying the potential for rapid and widespread transmission of contaminants and foodborne pathogens [1]. Beyond direct contamination, food quality is also compromised by spoilage, which is a major contributor to food waste. It is estimated that approximately one-third of all food produced for human consumption is lost or wasted annually, with spoilage being a leading cause in both developing and developed nations [1]. This not only represents a significant loss of resources but also raises ethical and environmental concerns. Consequently, the development of robust, sensitive, and rapid methods for monitoring food contaminants and quality indicators is of utmost importance to prevent, control, and mitigate the impact of potential outbreaks and spoilage [5,6].

### 1.2. Limitations of Conventional Technologies

Traditional laboratory methods for food analysis, such as high-performance liquid chromatography (HPLC) and gas chromatography-mass spectrometry (GC-MS), are well-established and serve as gold standards for accuracy and reliability [7]. However, these techniques suffer from significant practical limitations when applied to the demands of a fast-paced, globalized food industry. Most of these conventional methods are inherently laborious, time-consuming, require expensive and bulky equipment, and necessitate operation by highly skilled laboratory personnel [2]. These drawbacks render them largely incompatible with the need for rapid, on-site, and high-throughput screening of food products [4]. Similarly, conventional food preservation and packaging technologies, while effective to an extent, often lack active functionalities, such as the ability to regulate ripening gases, release antimicrobial agents on demand, or provide real-time feedback on food freshness [8]. These limitations create a critical gap in our ability to proactively manage food safety and quality throughout the supply chain.

### 1.3. Metal–Organic Frameworks (MOFs) as a Universal Platform

To overcome the shortcomings of conventional technologies, the scientific community has increasingly focused on advanced functional materials. Among these, metal–organic frameworks (MOFs) have emerged as an exceptionally promising and universal class of materials. MOFs are porous, crystalline coordination polymers constructed from metal ions or clusters (nodes) linked together by organic ligands (linkers) [9]. This unique “building block” approach allows for the rational design and synthesis of frameworks with precisely controlled and highly tunable properties [10].

MOFs are distinguished by a set of extraordinary characteristics that make them ideal candidates for addressing challenges in food safety and quality. These include: (1) exceptionally high specific surface areas and porosities, providing an abundance of active sites for adsorption and reaction; (2) tunable pore sizes and chemical environments, enabling high selectivity for specific target molecules; (3) uniform and well-defined crystalline structures, ensuring reproducibility; and (4) an easily functionalizable surface, which allows for their modification with specific recognition elements or catalytic sites [2,11]. Owing to this unparalleled versatility, MOFs have already demonstrated significant potential in diverse fields such as gas storage, separation, and catalysis [12]. Their application in sensing, in particular, has garnered significant attention, with novel MOF-based electrochemical sensors being developed for everything from hazardous ions to critical biomarkers [13,14]. Their application in the food science domain is a rapidly advancing frontier, offering innovative solutions for everything from contaminant detection to intelligent packaging.

### 1.4. Scope of This Review

This review provides a comprehensive and critical overview of the recent advancements in the application of multifunctional MOFs for enhancing food safety and quality. We aim to bridge the gap between materials science and food science, highlighting how the unique properties of MOFs can be harnessed to create next-generation technologies for the food industry. We begin by discussing the application of MOFs in advanced food analysis and contaminant detection, covering their roles in sample preparation, as chromatographic separation media, and as the core components of novel sensing platforms. Subsequently, the review explores the use of MOFs in food preservation and packaging, detailing their functions in active and intelligent packaging systems to control ripening, inhibit microbial growth, and indicate spoilage. Furthermore, we examine the emerging role of MOFs in food processing and decontamination, particularly for the adsorptive removal of contaminants from complex food matrices. Finally, the review concludes with a critical discussion on the toxicological concerns, current challenges—including stability, scalability, and cost—and future perspectives, providing an outlook on the transition of MOF-based technologies from laboratory research to practical application in the global food supply chain.

While several excellent reviews have previously covered specific aspects of MOFs in food science [1,15,16,17], they have often focused on a singular domain, such as their role as analytical tools for contaminant sensing or their application in food packaging. The novelty of this review lies in its comprehensive, integrated approach. We aim to provide a holistic “farm-to-table” perspective by connecting the disparate applications of MOFs across the entire food supply chain—from advanced analysis and sample preparation to active packaging, direct food processing, and decontamination. Furthermore, this review places a strong emphasis on the translational challenges and future perspectives, critically evaluating the hurdles of biocompatibility, scalability, cost, and regulatory approval that must be overcome for real-world implementation. By bridging these distinct research areas and focusing on the path to practical application, we intend to provide a unique and valuable resource for both materials scientists and food technology experts.

## 2. MOFs for Advanced Food Analysis and Contaminant Detection

The accurate and rapid detection of contaminants is a cornerstone of a robust food safety framework. Conventional analytical workflows, while precise, are often hindered by complex sample matrices and the ultra-trace concentrations of target analytes, necessitating laborious and time-consuming sample preparation steps [18]. MOFs have emerged as transformative materials in analytical chemistry, offering unprecedented opportunities to enhance nearly every stage of the analytical process. Their exceptional porosity, vast surface area, and highly tunable chemical functionalities make them superior candidates for sample preconcentration, chromatographic separation, and signal transduction in sensing platforms [19]. This section will comprehensively review the application of MOFs in revolutionizing food analysis and contaminant detection.

### 2.1. MOFs in Sample Preparation and Preconcentration

Sample preparation is frequently the most critical and challenging step in food analysis, as it aims to isolate and enrich target contaminants from complex matrices—such as fats, proteins, and sugars—that can interfere with subsequent detection [20]. MOFs, acting as advanced sorbents, have demonstrated exceptional performance in various extraction techniques due to their high adsorption capacities and selective recognition abilities, which can be tailored through pore engineering and post-synthetic modification.

Solid-phase extraction (SPE) is a widely used technique for sample cleanup and preconcentration. The performance of SPE is critically dependent on the choice of the sorbent material. MOFs have been extensively investigated as SPE sorbents, significantly outperforming traditional materials like silica gel or activated carbon. The high surface area of MOFs allows for greater loading capacity, while their well-defined pore structures and diverse functionalities (e.g., π-π stacking, hydrogen bonding, coordination interactions) enable selective capture of specific analytes [21]. For example, the highly stable chromium-based MOF, MIL-101(Cr), with its large pores and surface area, has been successfully employed for the extraction of sulfonamide antibiotics from water samples and mycotoxins from food matrices [22,23]. Similarly, Zeolitic Imidazolate Framework-8 (ZIF-8) has been utilized as an effective sorbent for the online SPE of tetracyclines from milk, attributed to its exceptional chemical stability and hydrophobic nature [24].

**Figure 1:**
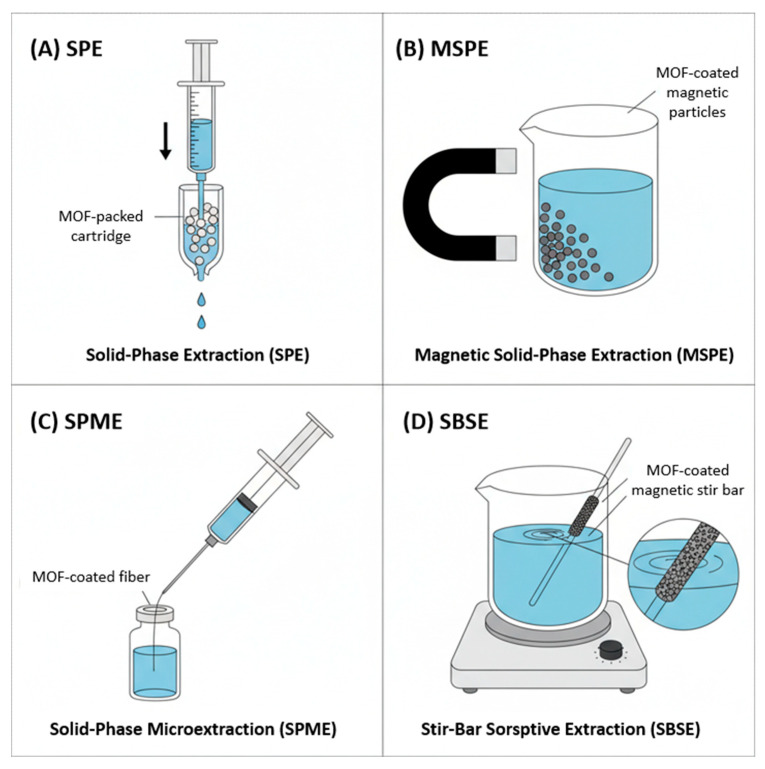
Schematic representation of key MOF-based sample preparation techniques. (**A**) Solid-Phase Extraction (SPE) using a MOF-packed cartridge. (**B**) Magnetic Solid-Phase Extraction (MSPE) where MOF-coated magnetic particles are collected by an external magnet. (**C**) Solid-Phase Microextraction (SPME) with a MOF-coated fiber. (**D**) Stir-Bar Sorptive Extraction (SBSE) using a MOF-coated magnetic stir bar.

To streamline the SPE process and eliminate the need for centrifugation or column packing, magnetic solid-phase extraction (MSPE) has been developed. In MSPE, magnetic nanoparticles (typically Fe_3_O_4_) are coated with a layer of sorbent material, allowing the entire composite to be easily manipulated with an external magnet (see Figure 1B). MOFs are ideal for this application, as they can be grown directly onto the surface of magnetic cores to form core–shell nanocomposites. These magnetic MOF composites combine the selective enrichment capabilities of the MOF shell with the convenient magnetic separability of the core. For instance, Fe_3_O_4_@ZIF-8 core–shell particles have been used for the MSPE of hormones from pork and fish, demonstrating high extraction efficiency and reusability [25]. The hierarchical structure and large surface area provided by the MOF shell facilitate rapid mass transfer and strong analyte interaction, leading to enhanced extraction performance (Table 1).

Solid-phase microextraction (SPME) and stir-bar sorptive extraction (SBSE) are solvent-free, non-exhaustive extraction techniques that integrate sampling, extraction, and concentration into a single step. In SPME, a thin fiber coated with a sorbent material is exposed to the sample (or its headspace), after which the adsorbed analytes are thermally or chemically desorbed into an analytical instrument (Figure 1C). MOFs have been developed as advanced SPME coatings, offering superior thermal stability and a much higher surface area compared to traditional coatings like polydimethylsiloxane (PDMS). For example, a MIL-53(Al) coated fiber demonstrated excellent performance for the extraction of polycyclic aromatic hydrocarbons (PAHs) from water, leveraging the framework’s π-π stacking interactions with the aromatic analytes [30].

SBSE is conceptually similar to SPME but utilizes a magnetic stir bar coated with a thick layer of sorbent (Figure 1D). This provides a significantly larger sorbent volume, leading to higher extraction capacity and lower detection limits. MOFs have been incorporated into SBSE coatings, often as a composite with PDMS, to create stir bars with enhanced selectivity and capacity. For example, a PDMS/IRMOF-3 coated stir bar was successfully used for the SBSE of estrogens from environmental water, demonstrating the benefit of the MOF’s specific interactions with the target molecules [31].

### 2.2. MOFs as Separation Media in Chromatography

The separation power of chromatographic techniques like gas chromatography (GC) and liquid chromatography (LC) is determined by the stationary phase. The uniform pore structures, high surface area, and tunable surface chemistry of MOFs make them excellent candidates for novel stationary phases with unique selectivity [1]. In GC, MOFs have been used as coatings for capillary columns. Their rigid frameworks and well-defined pore apertures enable size- and shape-selective separation of volatile and semi-volatile compounds. For instance, a MIL-101 coated capillary column achieved high-resolution separation of xylene isomers and ethylbenzene, a task that is challenging for conventional columns [32]. In LC, MOFs packed into columns have shown distinct separation mechanisms based not only on hydrophobicity but also on π-π interactions, hydrogen bonding, and coordination with open metal sites. MIL-53(Al), for example, has been used as an LC stationary phase for the successful separation of various aromatic positional isomers [33].

### 2.3. MOFs-Based Sensing Platforms

The development of rapid, sensitive, and portable sensors is crucial for on-site food safety monitoring. MOFs provide an ideal platform for constructing advanced sensors by acting as both a recognition element and a signal transducer. Their porous nature facilitates the preconcentration of analytes near the sensing interface, amplifying the signal, while their diverse compositions allow for a variety of signal output mechanisms [2].

Luminescent MOFs (LMOFs) are one of the most extensively studied classes of MOF-based sensors. Their sensing mechanism relies on changes in their fluorescence or phosphorescence intensity (quenching or enhancement) upon interaction with a target analyte. This luminescence can originate from the organic linkers (ligand-based), from encapsulated guest molecules, or from the metal nodes (e.g., lanthanide ions). The porous structure of MOFs enhances sensitivity by enriching analytes within the framework, maximizing their interaction with the luminescent centers [34]. A notable example is the use of an amino-functionalized aluminum MOF (NH_2_-MIL-53) for the highly sensitive detection of tetracycline antibiotics in milk. The tetracycline molecules quench the MOF’s intrinsic fluorescence through a combination of photoinduced electron transfer (PET) and inner filter effect (IFE) mechanisms [35].

Colorimetric sensors offer the advantage of visual detection without the need for sophisticated instrumentation. MOFs have been utilized in colorimetric assays primarily through their intrinsic enzyme-mimicking (nanozyme) activities. For example, many Cu-based and Fe-based MOFs exhibit peroxidase-like activity, catalyzing the oxidation of a chromogenic substrate (e.g., 3,3′,5,5′-tetramethylbenzidine, TMB) to produce a colored product. This reaction can be inhibited or promoted by a target analyte, leading to a visible color change. This principle has been applied to develop a colorimetric assay for E. coli, where aptamer-functionalized Cu-MOF nanoparticles serve as the peroxidase mimic in a sandwich-type immunoassay [36].

Electrochemical sensors detect analytes by measuring changes in electrical signals (current, potential, impedance). While most MOFs are poor electrical conductors, this limitation has been overcome by creating composites with conductive materials (e.g., graphene, carbon nanotubes) or by synthesizing inherently conductive MOFs. MOFs enhance electrochemical sensors by providing a high-surface-area scaffold for immobilizing recognition elements (e.g., antibodies, aptamers) and by offering electrocatalytic activity toward the target analyte [2]. For instance, an electrochemical sensor for the antibiotic tobramycin was fabricated using a nanocomposite derived from a bimetallic Ce/Cu-MOF, which provided high conductivity, excellent bio-affinity for the aptamer, and catalytic sites for signal enhancement [37].

Electrochemiluminescence (ECL) is a highly sensitive technique where light is generated at an electrode surface from electrochemically triggered reactions. MOFs have been used to construct advanced ECL sensors by acting as a stable matrix to load and concentrate ECL luminophores (such as Ru(bpy)_3_^2+^) and co-reactants, thereby amplifying the ECL signal. A Ru(bpy)_3_^2+^-functionalized MOF, for example, was developed for the highly sensitive detection of melamine in milk, demonstrating significantly enhanced ECL intensity and stability compared to the free luminophore [38].

Surface-Enhanced Raman Scattering (SERS) is an ultra-sensitive vibrational spectroscopy technique that can provide fingerprint-like information for molecular identification. The SERS signal is dramatically amplified when a molecule is adsorbed on or very close to a plasmonic nanostructure (typically Au or Ag), known as a “hot spot.” MOFs have been ingeniously integrated with plasmonic nanoparticles to create highly effective SERS substrates. In these composites, the MOF shell acts as a molecular sieve to selectively capture and enrich target analytes from a complex sample, while simultaneously bringing them into close proximity with the plasmonic core. This dual function not only enhances sensitivity and selectivity but also prevents the aggregation of the nanoparticles, improving signal stability and reproducibility. A tailored necklace-like Ag@ZIF-8 core–shell heterostructure was shown to be an excellent SERS platform for the ultrasensitive detection of the fungicide thiram and the illegal additive melamine [39].

It is valuable to contextualize MOF-based sensors against alternative approaches. In the realm of SERS, the engineered MOF@plasmonic core heterostructures offer exceptional selectivity through their molecular sieving effect. However, this performance often relies on complex, multi-step syntheses. A contrasting and more sustainable strategy involves leveraging naturally derived biotemplates to fabricate SERS substrates. For instance, recent research has demonstrated that bio-waste, such as the eggshells of the Chinese oak silkworm, can serve as effective, low-cost templates for depositing plasmonic nanostructures to create SERS-active surfaces [40]. While such bio-inspired materials present clear advantages in terms of cost and sustainability, they typically lack the rationally designed molecular selectivity that is the hallmark of MOFs. Ultimately, the choice of sensing platform will depend on the specific application’s required balance between selectivity, sensitivity, cost, and manufacturability.

## 3. MOFs for Food Preservation and Packaging

Food packaging is an indispensable component of the modern food supply chain, serving the primary function of protecting food products from physical damage, microbial contamination, and detrimental environmental factors such as oxygen, moisture, and light [1]. Traditional packaging materials act as passive barriers. However, the increasing demand for longer shelf life, reduced food waste, and enhanced consumer safety has spurred the development of advanced packaging systems, namely active and intelligent packaging [41]. MOFs, with their vast internal surface areas, tunable porosities, and capacity for encapsulating guest molecules, are uniquely suited to revolutionize this field. They can be integrated into packaging systems to actively modulate the package atmosphere, release beneficial agents, indicate food quality, and enhance barrier properties.

### 3.1. Active Packaging: Gas Regulation and Antimicrobial Release

Active packaging goes beyond passive containment by actively interacting with the food or its surrounding environment to improve quality and extend shelf life. MOFs can be engineered to perform specific active functions, most notably the regulation of gases critical to ripening and the controlled release of antimicrobial compounds to inhibit spoilage.

Ethylene (C_2_H_4_) is a gaseous plant hormone that plays a crucial role in initiating and accelerating the ripening and subsequent senescence of climacteric fruits and vegetables, such as bananas, tomatoes, and avocados [42]. The accumulation of ethylene within a sealed package can drastically shorten the product’s shelf life. Consequently, scavenging ethylene from the package headspace is a highly effective strategy for ripening control. While traditional scavengers like potassium permanganate (KMnO_4_) are used, they can pose safety concerns due to potential migration into the food.

MOFs present a superior alternative for ethylene scavenging due to their high adsorption capacity and selectivity. The capture of ethylene by MOFs can occur through two primary mechanisms: (1) physisorption within the porous network and (2) selective chemisorption via π-complexation between the ethylene double bond and unsaturated metal sites within the MOF structure [1]. MOFs containing open metal sites, particularly Cu(II) or Ag(I), have shown exceptional affinity for ethylene. For instance, the commercially available copper-based MOF, Basolite C300 (also known as HKUST-1), was investigated for its ability to adsorb ethylene, demonstrating significant potential for extending the shelf life of fresh produce [43]. In another study, a copper terephthalate MOF (CuTPA) was shown to effectively encapsulate ethylene, allowing for its controlled removal from the environment to slow the ripening of bananas [44]. These studies highlight the potential of incorporating MOFs into packaging films or sachets as highly efficient and safe ethylene scavengers.

Microbial contamination by bacteria, yeasts, and molds is a leading cause of food spoilage and foodborne illness. Incorporating antimicrobial agents into packaging materials is an effective strategy to inhibit microbial growth on the food surface. The key challenge lies in controlling the release of these agents, ideally in response to conditions that favor microbial proliferation, such as high humidity. MOFs serve as excellent nanocarriers for encapsulating a wide range of antimicrobial compounds, protecting them from degradation and enabling their stimulus-responsive release.

Natural antimicrobial agents, such as essential oils and their active components (e.g., allyl isothiocyanate (AITC), carvacrol, thymol), are highly desirable due to consumer preference for natural preservatives. However, these compounds are often volatile and unstable. Lashkari et al. [45] demonstrated the encapsulation of AITC within the pores of three different MOFs (HKUST-1, MOF-74(Zn), and RPM6-Zn). The release of AITC was triggered by high relative humidity (95–100%), as water molecules displaced the encapsulated AITC from the MOF pores. This humidity-triggered release is a particularly “smart” mechanism, as high moisture levels are a prerequisite for most microbial growth. Beyond acting as carriers, some MOFs possess intrinsic antimicrobial properties. The slow release of metal ions, such as Ag^+^ or Co^2+^, from MOF structures has been shown to exert potent bactericidal effects. For example, cobalt-based zeolitic imidazolate framework (ZIF-67) and various silver-based MOFs have demonstrated significant activity against common foodborne pathogens like E. coli and S. aureus [46,47].

### 3.2. Intelligent Packaging: Spoilage and Freshness Indication

Intelligent packaging is designed to monitor the condition of the packaged food and provide real-time, easily interpretable information about its quality and freshness. This is typically achieved by detecting specific chemical or microbial changes associated with spoilage. MOFs, particularly luminescent MOFs (LMOFs), are ideal candidates for fabricating such sensor systems. They can be engineered to exhibit a distinct optical response (colorimetric or fluorometric) upon interaction with target spoilage indicators, such as biogenic amines (e.g., histamine, cadaverine, putrescine), pH changes, or spoilage-related gases (NH_3_, H_2_S).

For example, Xu et al. [48] developed a sophisticated sensor based on a lanthanide MOF composite (MR@EuMOFs) that could detect histamine, a key indicator of spoilage in fish. The sensor exhibited a ratiometric fluorescent response that could be visually interpreted, providing a clear signal of food deterioration. More recently, MOF-based Surface-Enhanced Raman Scattering (SERS) platforms have been designed for freshness indication. Kim et al. [49] fabricated a flexible SERS paper by coating a ZIF-8 layer onto a Au nanoparticle-impregnated substrate. The ZIF-8 layer served to capture and concentrate the volatile biogenic amines putrescine and cadaverine, allowing for their ultra-sensitive detection and providing a quantitative measure of meat spoilage. These MOF-based indicators can be integrated into packaging labels as disposable sensors, empowering consumers and retailers to make more informed decisions about food safety.

### 3.3. Barrier Properties Enhancement in Packaging Materials

The primary function of any food packaging is to act as a barrier to the permeation of gases and water vapor. Oxygen promotes oxidative degradation of lipids and vitamins and supports the growth of aerobic microbes, while moisture can lead to microbial growth, texture changes, and caking in dry foods. Polymers are widely used in packaging but often have insufficient barrier properties. One effective strategy to enhance these properties is to create mixed-matrix membranes (MMMs) by incorporating impermeable fillers into the polymer matrix.

**Figure 2:**
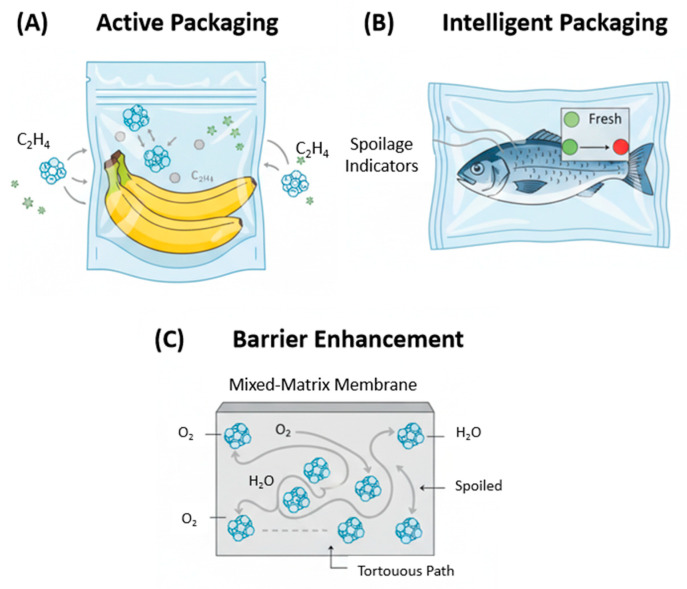
Multifunctional roles of MOFs in advanced food packaging systems. (**A**) Active Packaging: MOFs integrated into the packaging scavenge detrimental gases like ethylene (C_2_H_4_) and provide controlled release of antimicrobial agents to inhibit spoilage. (**B**) Intelligent Packaging: A MOF-based sensor patch embedded in the packaging material provides a visual signal (e.g., color change; the red color indicates deterioration) by detecting spoilage volatile compounds (e.g., biogenic amines), indicating the freshness of the product. (**C**) Barrier Enhancement: The incorporation of MOF nanoparticles into a polymer matrix creates a mixed-matrix membrane (MMM) with a more tortuous diffusion pathway, significantly reducing the permeation of oxygen and water vapor.

MOF nanoparticles are excellent fillers for this purpose. When dispersed within a polymer, they force permeating gas or water molecules to follow a longer, more tortuous diffusion pathway around the impermeable crystals, thereby significantly reducing the overall permeability of the film (Figure 2C). The choice of MOF is critical; hydrophobic and water-stable MOFs are particularly effective for creating water vapor barriers. For instance, Bae et al. [50] demonstrated that incorporating the highly water-stable zirconium-based MOF, MOF-801, into a hydrophobic polymer resulted in a composite film with dramatically enhanced water vapor barrier properties. Similarly, ZIF-8 nanoparticles have been incorporated into biopolymer films like chitosan, not only improving the film’s mechanical strength and water resistance but also imparting antimicrobial activity [51]. This multifunctional approach, where MOFs simultaneously enhance barrier, mechanical, and active properties, represents a significant advancement in food packaging technology.

### 3.4. Methods for Incorporating MOFs into Packaging Materials

The practical application of MOFs in food packaging relies on effectively integrating them into packaging materials without compromising the material’s integrity or safety [52]. Several methods have been developed to achieve this:

Mixed-Matrix Membranes (MMMs): This is the most common approach, where MOF nanoparticles are dispersed as a filler within a polymer matrix (e.g., chitosan, PLA, polyethylene) before the film is cast or extruded [53]. As discussed in Section 3.3, this method is highly effective for enhancing barrier properties. The key challenges are ensuring uniform dispersion of the MOFs and maintaining strong adhesion between the MOF particles and the polymer to avoid defects.

Surface Coating: MOFs can be coated onto the surface of a packaging film [54]. This can be achieved through various techniques such as dip-coating, spray-coating, or layer-by-layer assembly. This method is particularly useful for creating sensor patches for intelligent packaging, where the MOF-based sensor is localized and can directly interact with the package headspace.

Sachets and Pads: For applications like ethylene scavenging or moisture control, MOFs can be enclosed within small, gas-permeable sachets or pads that are placed inside the food package [54]. This method avoids direct contact between the MOF and the food product, which can simplify regulatory approval and is a simple way to retrofit existing packaging systems. The design of the sachet material is crucial to ensure it is robust and allows for efficient gas exchange.

## 4. MOFs for Food Processing and Decontamination

Beyond their roles in analysis and packaging, MOFs are emerging as powerful tools for direct intervention within food matrices, offering novel solutions for processing and decontamination. In this context, MOFs can act as highly selective “nanosponges” to remove unwanted compounds or as robust platforms for catalysis to facilitate desirable transformations. This direct application within food processing streams necessitates the use of MOFs that are not only highly efficient but also stable under processing conditions (e.g., in aqueous or oily media) and, crucially, biocompatible. This section explores the dual application of MOFs as advanced adsorbents and heterogeneous catalysts for improving food quality and safety.

### 4.1. Adsorptive Removal of Contaminants from Food Matrices

**Figure 3:**
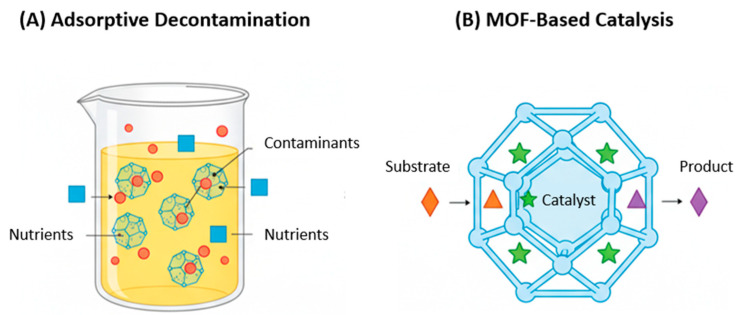
Schematic illustration of MOF applications in direct food processing and decontamination. (**A**) Adsorptive Decontamination: MOFs with tailored pore apertures act as selective adsorbents, capturing specific contaminants (red circles) from a liquid food matrix while allowing desirable components like nutrients and flavors (blue squares) to remain. (**B**) MOF-based Catalysis: MOFs serve as robust hosts for catalysts (e.g., enzymes or acids, shown as green stars). The porous framework protects the catalyst and allows substrate molecules (orange triangles) to diffuse in and react, after which the desired product molecules (purple diamonds) diffuse out, enabling easy separation and catalyst reuse.

Many raw food materials, such as crude oils and fruit juices, can contain undesirable substances that compromise their quality, safety, and shelf life. These can include natural degradation products, environmental contaminants, or toxins. Traditional purification methods often involve energy-intensive processes or non-selective adsorbents that can also remove beneficial compounds like flavors and nutrients. MOFs, with their precisely tailored pore environments and enormous surface areas, offer a far more selective and efficient means of decontamination (Figure 3A).

The purification of edible oils is a prime example. Crude vegetable oils contain free fatty acids (FFAs) and peroxide compounds that lead to rancidity and off-flavors. Vlasova et al. [55] demonstrated that MOFs based on biocompatible metals, such as Ti-MOF, could effectively reduce the acid and peroxide values of unrefined sunflower oil. The MOFs acted as solid adsorbents, selectively trapping the polar FFAs and peroxides within their porous structure while leaving the desirable triglyceride molecules untouched. Importantly, the MOFs could be easily regenerated by a simple solvent wash and reused multiple times, highlighting the sustainability of the process.

In the beverage industry, MOFs have shown great promise for removing specific toxins. Patulin, a mycotoxin produced by certain molds, is a common contaminant in apple juice and a significant food safety concern. Conventional removal using activated carbon is often non-selective. Addressing this, Liu et al. [56] developed a novel adsorbent by functionalizing a highly stable zirconium-based MOF, UiO-66-NH_2_, with cysteine. This composite material exhibited exceptionally high and selective adsorption of patulin from apple juice, reducing its concentration to levels well below the limit recommended by the World Health Organization (WHO). The high efficiency was attributed to the abundant active sites (amine, carboxyl, and sulfhydryl groups) that formed specific hydrogen bonding and coordination interactions with the patulin molecule. Furthermore, MOFs like UiO-67 have been successfully used to remove illegal food dyes, such as Congo red and Malachite green, from aqueous solutions with remarkable adsorption capacities, demonstrating their potential for cleaning up contaminated liquid foods [57].

### 4.2. MOFs as Catalysts in Food Processing

The use of catalysts is fundamental to many modern food processing operations, enabling reactions that improve texture, flavor, or nutritional value. Most industrial processes rely on homogeneous catalysts (dissolved in the reaction medium), which are difficult and costly to separate from the final food product. MOFs offer a paradigm shift by serving as highly stable and recyclable heterogeneous catalysts (Figure 3B). Their catalytic activity can be intrinsic to the framework’s metal nodes and linkers, or they can act as protective hosts for other catalytic species like enzymes or molecular catalysts.

A compelling example of MOFs’ catalytic potential is in the production of low-calorie structured lipids. Xie et al. [58] demonstrated an innovative approach by encapsulating a Keggin-type solid acid catalyst (Cs_2.5_H_0.5_PW_12_O_40_) within the pores of the exceptionally stable MOF, UiO-66. This composite material was then used to catalyze the acidolysis of soybean oil. The MOF served multiple functions: it stabilized the acid catalyst, prevented its leaching into the oil, and its defined pores provided a shape-selective environment for the reaction, leading to a high yield of the desired low-calorie product. After the reaction, the solid MOF catalyst could be easily recovered by simple filtration and reused.

Furthermore, MOFs are being explored as superior scaffolds for enzyme immobilization. Enzymes are highly efficient biocatalysts used in food processing (e.g., lactase to produce lactose-free dairy), but they are often fragile and lose activity under operational conditions [59]. Encapsulating enzymes within the pores of a MOF—a process known as “biomimetic mineralization”—can physically protect them from denaturation by heat, pH changes, or organic solvents. This “ship-in-a-bottle” approach not only enhances enzyme stability and longevity but also creates a reusable solid biocatalyst, simplifying downstream processing and reducing costs. The tunable pore environment of MOFs can also influence the enzyme’s activity and selectivity, opening avenues for creating highly specialized biocatalytic systems for the food industry.

### 4.3. Encapsulation and Controlled Release of Nutraceuticals

Beyond removing contaminants, MOFs offer exciting potential as protective nanocarriers to enhance the nutritional value of food products. Many valuable nutrients and nutraceuticals, such as vitamins, antioxidants (e.g., curcumin, resveratrol), and essential fatty acids, are highly sensitive to light, oxygen, and heat, leading to significant degradation during processing and storage. MOFs can encapsulate these delicate molecules within their pores, shielding them from harsh environmental conditions [60].

For instance, cyclodextrin-based MOFs (CD-MOFs), which are constructed from biocompatible building blocks, have been shown to be excellent hosts for encapsulating curcumin, a potent but unstable antioxidant. The MOF not only enhanced the stability of the curcumin but also facilitated its controlled release under simulated gastrointestinal conditions [61]. Similarly, research has demonstrated the potential of stable, biocompatible MOFs like ZIF-8 to encapsulate and protect Vitamin C (ascorbic acid), a notoriously unstable nutrient [62]. The high porosity of MOFs allows for significant loading capacities, while the tunable nature of the framework can be engineered to trigger the release of the nutrient in response to specific stimuli, such as a pH change in the digestive tract. This application opens a new frontier for MOFs in the development of functional foods and fortified products with improved bioavailability and shelf-life [63].

### 4.4. Selection of MOFs Based on Structure-Functionality

The selection of a specific MOF is critical and is dictated by its inherent structural and chemical properties. A direct comparison of different MOF platforms reveals clear structure-activity relationships that govern their suitability for a given task, as summarized in Table 2. For example, the exceptional chemical and thermal stability of the zirconium-based UiO-66, derived from its strong Zr-O clusters, makes it a superior candidate for applications in harsh aqueous or acidic environments, such as pesticide adsorption from fruit juices [64]. In contrast, the dynamic and pH-sensitive nature of the zinc-based ZIF-8 framework, while less robust, is highly advantageous for creating “smart” systems where controlled release of an active agent is triggered by changes in local acidity, a common signal of microbial growth [65]. Meanwhile, the open metal sites in copper-based HKUST-1 provide high affinity for specific molecules like ethylene, making it a premier choice for active packaging to delay fruit ripening [66].

## 5. Toxicology, Challenges, and Future Perspectives

While the preceding sections have highlighted the immense potential and functional advantages of MOFs in enhancing food safety and quality, the transition from laboratory proof-of-concept to widespread industrial application is contingent upon overcoming several significant hurdles. This final section provides a critical assessment of the toxicological concerns associated with MOF usage, outlines the current technical and economic challenges, and offers a perspective on the future trajectory of this exciting field. A responsible and forward-thinking approach requires a balanced view of both the promise and the perils.

### 5.1. Biocompatibility and Toxicological Concerns of MOFs

The foremost concern for any material intended for direct or indirect contact with food is its safety for human consumption. For MOFs, toxicological considerations are paramount and can be broadly categorized into the potential risks from the constituent components and the material’s nanoparticle form.

The primary toxicological risk stems from the potential leaching of the MOF’s building blocks—metal ions and organic linkers—into the food matrix. Many MOFs are synthesized using metals that could be toxic at certain concentrations, such as chromium (in MIL-series MOFs) or copper (in HKUST-1) [1]. Similarly, many organic linkers are synthetic aromatic compounds whose metabolic fate and long-term effects upon ingestion are not well understood. Therefore, a critical area of research is the development and use of MOFs constructed from biocompatible and “Generally Recognized as Safe” (GRAS) components. Significant progress has been made in this area with the development of “bio-MOFs” using metals like Fe(III), Zr(IV), Ca(II), and Mg(II), and linkers derived from natural products such as amino acids, peptides, nucleobases, and cyclodextrins (e.g., CD-MOFs) [67]. Zirconium-based MOFs, particularly the UiO-66 family, are often highlighted as leading candidates due to the exceptionally high stability of the Zr-O cluster, which minimizes metal leaching, and the relatively low toxicity profile of zirconium [1].

Beyond chemical composition, the physical form of MOFs, often as nanoparticles, presents another layer of toxicological complexity. The field of nanotoxicology has shown that the size, shape, and surface chemistry of nanoparticles can significantly influence their interaction with biological systems, including their absorption, distribution, metabolism, and excretion (ADME) profile. Comprehensive studies on the behavior of MOF nanoparticles in the gastrointestinal tract, their potential for bioaccumulation, and their long-term systemic effects are still in their infancy and are urgently needed before regulatory approval for food applications can be considered.

### 5.2. Current Challenges: Stability, Scalability, and Cost

For MOF-based technologies to be practically viable, they must overcome three major interconnected challenges: chemical stability, manufacturing scalability, and economic cost.

Stability: Food systems are notoriously complex and harsh environments, often aqueous and containing a wide variety of salts, acids, and enzymes. Many early and highly porous MOFs (e.g., MOF-5) exhibit poor hydrolytic stability, readily degrading in the presence of moisture [2]. This severely limits their application in most food-related scenarios. As mentioned, the development of water-stable frameworks, such as the Zr-based UiO series, has been a major breakthrough. However, long-term stability under real-world food processing and storage conditions—which may involve temperature fluctuations, pH changes, and exposure to oxidative environments—remains a critical benchmark that must be rigorously validated for each specific application.

Scalability and Cost: The majority of MOF syntheses reported in the literature are small-scale laboratory procedures that often rely on solvothermal methods using large volumes of toxic organic solvents (like DMF), expensive organic linkers, and energy-intensive high-temperature reactions. These methods are neither environmentally sustainable nor economically feasible for the large-scale production required for widespread use in the food industry [1]. The future of MOF manufacturing hinges on the development of green and scalable synthesis routes. As comprehensively reviewed by Kumar et al. [68], a range of promising alternatives to conventional solvothermal methods are emerging. These include mechanochemical synthesis (solvent-free grinding), sonochemical and microwave-assisted methods, and continuous flow reactions, all of which can dramatically reduce reaction times and energy consumption. Furthermore, transitioning to aqueous-based syntheses at ambient temperature and pressure is a critical goal. These methods not only reduce or eliminate the reliance on toxic and expensive organic solvents like DMF but also simplify downstream purification processes, directly addressing the environmental, safety, and economic hurdles that currently impede commercialization. Furthermore, the cost of the organic linkers remains a significant barrier. Shifting focus to MOFs built from inexpensive, readily available linkers derived from biomass or industrial feedstocks will be crucial for their commercial viability.

### 5.3. Future Outlook: From Laboratory Research to Practical Application

The field of MOFs in food science is at an exciting inflection point, poised to move from fundamental research toward real-world problem-solving. To accelerate this transition, future efforts should be focused on the following strategic priorities:

Establishing a “Food-Grade” MOF Library: The highest priority is to expand the range of MOFs built exclusively from Generally Recognized as Safe (GRAS) components (e.g., iron, zinc, calcium; linkers like citrate, succinate, amino acids). A key research goal should be to perform standardized stability and leaching tests on these “bio-MOFs” under simulated food processing and digestion conditions, aiming for metal and linker leaching levels that are well below the established tolerable daily intake limits.

Developing Green, Scalable Synthesis Protocols: The future of MOF manufacturing hinges on moving away from solvothermal methods. Research should prioritize water-based synthesis, mechanochemistry (solvent-free grinding), and continuous flow processes. A critical benchmark for success will be the development of protocols capable of producing kilograms of stable, high-quality MOFs with a space-time yield and production cost that are competitive with traditional adsorbents like activated carbon.

Designing and Testing Integrated Multifunctional Systems: The true potential of MOFs lies in multifunctionality. Future work should move beyond single-purpose materials to design smart packaging films that, for example, simultaneously scavenge ethylene, release humidity-triggered antimicrobials, and provide a clear colorimetric signal for spoilage. The goal is to create integrated systems where multiple functions are performed by a single, efficiently designed composite material.

Rigorous Prototyping and Real-World Validation: A concerted effort is needed to bridge the gap between laboratory results and industrial application. This involves fabricating and testing MOF-integrated prototypes (e.g., sensor labels, active packaging films, filtration cartridges) with real food products and under realistic storage and transport conditions. This will require deeply collaborative efforts between materials chemists, food scientists, toxicologists, and industry stakeholders to co-develop solutions that are practical, safe, and effective.

## 6. Conclusions

In conclusion, this review has comprehensively surveyed the vast and rapidly expanding landscape of multifunctional metal–organic frameworks applied to the critical challenges of food safety and quality. The unique and highly tunable properties of MOFs—including their exceptional porosity, vast surface area, and chemical versatility—position them as a transformative class of materials. In food analysis, they have demonstrated the ability to dramatically enhance sample preparation efficiency and create sensing platforms with unprecedented sensitivity and selectivity. In food preservation and packaging, they are enabling the development of next-generation active and intelligent systems that can extend shelf life and provide real-time quality feedback. Furthermore, in food processing, MOFs are offering sustainable solutions for selective decontamination and robust heterogeneous catalysis.

Despite this remarkable potential, significant hurdles remain on the path to widespread adoption. The paramount concerns of biocompatibility and long-term toxicology must be addressed through rigorous, standardized evaluation and the rational design of MOFs from inherently safe building blocks. Concurrently, the formidable challenges of chemical stability in complex food matrices and the development of scalable, cost-effective, and environmentally benign synthesis methods must be overcome to ensure commercial viability.

The journey of MOFs from the chemistry laboratory to the consumer’s table is still underway, but the progress to date is a clear harbinger of their disruptive potential. By fostering deep and sustained collaboration across scientific and industrial disciplines, the promise of MOFs to help secure a safer, higher-quality, and more sustainable global food supply can be fully realized, marking a new era in the application of advanced materials for human well-being.

## Figures and Tables

**Table 1:** Representative applications of MOF-based materials in sample preparation for food analysis.

MOF Material	Target Analyte(s)	Food Matrix	Method	Reference
MIL-101(Cr)	Sulfonamides (e.g., SMZ, SCP)	Water	SPE, DSPE	[22]
CD-MOFs-1	Sulfonamides (e.g., STZ, SMR)	Chicken, Pork	SPE	[26]
Fe_3_O_4_@ZIF-8@MIP	Hormones (e.g., E_1_, E_2_)	Pork, Fish	MSPE	[25]
MIP/Au@Cu-MOF/ N-GQDs	Mycotoxin (Patulin)	Apple Juice	MSPE	[23]
MIL-101(Cr)@MIP	Pesticides (Trichlorfon)	Pear, Apple	SPE, DSPE	[27]
ZIF-90 derived NPC	Pyrethroid pesticides	Peach, Cucumber	SPME	[28]
MIL-101-Cr-NH_2_	Organophosphorus pesticides	Water	SBSE	[29]

Note: SMZ, Sulfamethazine; SCP, Sulfachloropyridazine; STZ, Sulfathiazole; SMR, Sulfamerazine; E_1_, Estrone; E_2_, Estradiol; MIP, Molecularly Imprinted Polymer; NPC, Nanoporous Carbon.

**Table 2:** Comparative analysis of representative MOFs in food safety and quality applications.

MOF Type	Key Structural Features	Example Application	Performance & Structure-Activity Relationship	Limitations
UiO-66 (Zr)	Zr-O clusters; high hydrolytic & thermal stability; microporous (~6 Å)	Pesticide Adsorption	The robust framework is stable in water and acidic media. Strong coordination between Zr clusters and functional groups on pesticides enables high adsorption efficiency.	Smaller pore size may limit uptake of larger molecules; synthesis often requires high temperatures.
ZIF-8 (Zn)	Zn-N bonds; sodalite topology; pH-responsive framework; hydrophobic	Antimicrobial Release	The framework is unstable at low pH, allowing for acid-triggered release of encapsulated antimicrobial agents or bactericidal Zn^2+^ ions.	Poor stability in acidic foods; potential for zinc leaching.
HKUST-1 (Cu)	Paddle-wheel Cu_2_ clusters; open metal sites; moderate water stability	Ethylene Scavenging	The unsaturated copper sites act as strong binding points for ethylene via π-complexation, leading to highly effective and selective removal from package headspace.	Limited stability in the presence of high moisture; potential for copper leaching.

## Data Availability

No new data were created or analyzed in this study. Data sharing is not applicable to this article.
